# The Importance of Solvent Effects on the Mechanism of the Pfeiffer Effect

**DOI:** 10.3390/inorganics6030087

**Published:** 2018-08-29

**Authors:** Jamie L. Lunkley, Ngoc M. Nguyen, Kristina M. Tuminaro, Dana Margittai, Gilles Muller

**Affiliations:** Department of Chemistry, San José State University, One Washington Square, San José, CA 95192-0101, USA; Jamie0767_wvceops@yahoo.com (J.L.L.); Department of Chemistry, San José State University, One Washington Square, San José, CA 95192-0101, USA; Ngoc1986@gmail.com (N.M.N.); Department of Chemistry, San José State University, One Washington Square, San José, CA 95192-0101, USA; kristina.tuminaro@gmail.com (K.M.T.); Department of Chemistry, San José State University, One Washington Square, San José, CA 95192-0101, USA; dmargittai@hotmail.com (D.M.); Department of Chemistry, San José State University, One Washington Square, San José, CA 95192-0101, USA

**Keywords:** circularly polarized luminescence, chirality transfer, hydrophobic interactions, D_3_ lanthanide(III) complexes, chiral amino acid, solvent packing

## Abstract

The Pfeiffer effect is observed when an optically active compound such as an amino acid is introduced to a solution containing a labile racemic metal complex, and an equilibrium shift is obtained. The “perturbation” results in an excess of one enantiomer over the other. The shift is a result of a preferential outer sphere interaction between the introduced chiral species and one enantiomeric form (Λ or Δ) of a labile metal complex. Speculations regarding the mechanism of the Pfeiffer effect have attributed observations to a singular factor such as pH, solvent polarity, or numerous other intermolecular interactions. Through the use of the lanthanide(III) complexes [Tb(DPA)_3_]^3−^ and [Eu(DPA)_3_]^3−^ (where DPA = 2,6-pyridinedicarboxylate) and the amino acids l-serine and l-proline; it is becoming clear that the mechanism is not so simply described as per the preliminary findings that are discussed in this study. It appears that the true mechanism is far more complicated than the attribute just a singular factor. This work attempts to shine light on the fact that understanding the behavior of the solvent environment may hypothetically be the key to offering a more detailed description of the mechanism.

## 1. Introduction

Chirality is an important phenomenon that occurs naturally in many of the biological mechanisms that are essential to life. The secondary structures of enzymes and proteins contain chiral amino acids. These amino acids are most often found within the active sites of biologically important enzymes. These active sites are what bind the incoming substrate. A substrate with the wrong “fit”, or more simply, the incorrect enantiomer of a substrate, can prove to be disastrous for the enzyme to which it binds. There is also the potential that the entire biological system as a whole is affected by this as well. The pharmaceutical industry is a prime example of this important relationship between the chirality of the host enzyme and the guest substrate. Knowing the chirality of a pharmaceutical drug with certainty, for instance, is important the drug is marketed to the public. If a chiral drug is marketed to the public without knowing with absolute certainty, its chirality could prove disastrous to both the company that marketed the drug, and to the public that consumes it. A classic example of this is that of thalidomide.

Thalidomide was originally introduced and prescribed to pregnant mothers to treat the symptoms of morning sickness. At the time that it was prescribed, it was not known that the drug was not a single enantiomer, but rather it was a racemic mixture of the *R*- and *S*-enantiomers of thalidomide. The unfortunate side effects of this were that children born to the mothers who were prescribed thalidomide suffered debilitating birth defects. The main effects caused by racemic thalidomide were congenital deformities that affected the proper development of the limbs. This included a bilateral deformity of either both arms or both legs; in more severe cases, all four limbs were affected. The eyes, ears, and cardiovascular systems could also be affected, depending upon the severity and the timing of exposure to thalidomide [1]. Thalidomide stands as the prime example for the importance of identifying with absolute certainty the chirality of modern pharmaceutical drugs. Most drugs on the market today have at least one chiral center [2,3]. The most common obstacle the pharmaceutical industry faces with the development of chiral drugs, is the identification or elucidation of enantiomers [2,3]. There are several methods that are available and that are commonly employed in the identification and elucidation of chiral drugs [3]. The most common include circular dichroism (CD) [4–7], high performance liquid chromatography (HPLC) [8–11], NMR spectroscopy [12–15], and X-ray crystallography [16–19], to cite a few. More recently, a technique referred to as the crystalline sponge method is beginning to show its potential for the elucidation of chiral molecules [20–22]. While useful, these methods are not without their challenges. X-ray crystallography, for instance, is the most commonly relied upon method for the structural elucidation of chiral molecules [16–19]. It is extremely valuable when identifying the chirality of molecules, but the challenge is obtaining pure and suitable crystals from often small amounts of material. This method can sometimes take weeks or months, in order to grow suitable enough crystals for study. It is often also difficult to obtain crystals from liquid media in some cases [16–19]. NMR spectroscopy is also often useful for the identification of chiral molecules; however, this technique often requires the use of expensive chiral shift reagents [12–15]. The NMR spectrum that results is often convoluted and difficult to interpret without additional methods to further identify the molecule in question [12–15].

The most recent technique is the crystalline sponge method, which is an interesting form of X-ray crystallography that was first introduced by Fujita et al. in 2015 [20]. This method is unique, as it essentially avoids the need for direct crystallization of a molecule [20,21]. Instead, the crystalline sponge method exploits porous metal-organic scaffolds such as ones composed of [(ZnI_2_)3(tpt)_2_(c-C_6_H_12_)_*x*_]_*n*_ (tpt = tris(4-pyridyl)-1,3,5-tri-azine) [20,21]. Molecules of interest are adsorbed by the crystalline sponge, and the target molecules arrange themselves into a uniform orientation within the sponge scaffold. When chiral molecules are introduced to the crystalline sponge, a shift in the space group (achiral to chiral) occurs. This method is gaining attention, not only due to its potential to aid in the confident identification of chiral molecules, but because relatively small sample sizes can be utilized. Though useful and promising, this method is not without its challenges. There are certain size constraints regarding the target molecules; if they are too large, they will not be absorbed through the pores of the crystalline sponge. There are also certain functional group constraints as well that will need to be addressed in order to better utilize this method of elucidation [20,21].

Despite there being a number of useful tools that are available to identify or assign absolute chirality to the molecules bearing chiral centers, there is still a need to develop additional techniques that can add further confidence as the field advances. Circularly polarized luminescence (CPL), which is an emission analog to the familiar CD spectroscopy, is one such technique that continues to gain interest in fields that utilize chiral probes [23–43]. CPL is a valuable tool that can be used in addition to the current elucidation methods to add further information and to aid in the identification of chiral molecules. In contrast to CD spectroscopy, which relays upon excitation methods (| *g*_*abs*_ |), the information gained by the use of CPL spectroscopic techniques are specific to only CPL active species present in a sample. The information gained by CD methods can often be from all chromophoric species present in the sample being studied, it is not specific to one target. CPL active species that exhibit long excited state lifetimes add the benefit of the collected data being free of potentially interfering background signals. In addition to the mentioned benefits, CPL is not concentration-dependent, and small amounts of a CPL active species are required to obtain sufficient data [23–43].

The Pfeiffer effect is one method that utilizes CPL spectroscopy to probe the chirality of biologically important molecules such as amino acids, as in this particular work. The Pfeiffer effect is observed when an optically active compound such as a chiral amino acid is introduced to a solution containing a racemic mixture of a labile metal complex. The optically active compound or CEC (chiral environment compound) that induces a shift in the racemic equilibrium of the metal complex which results in one of the complex enantiomers being in abundance over the other [24,27,28]. The Pfeiffer effect was first observed and reported by P. Pfeiffer and coworkers with transition metal complexes [44–47], In recent years the Pfeiffer effect has been observed with lanthanide(III)-based complexes containing achiral ligands such as those with DPA (2,6-pyridinedicarboxylate) or derivatives [24,27,28]. Lanthanide(III)-based complexes with DPA or similar derivatives with either Eu^3+^ or Tb^3+^ at the core of the complex [24,27,28,48–51]. These complexes possess D_3_ symmetry; D_3_ refers to the point group to which these types of complexes are assigned [51].

**Figure 1.**
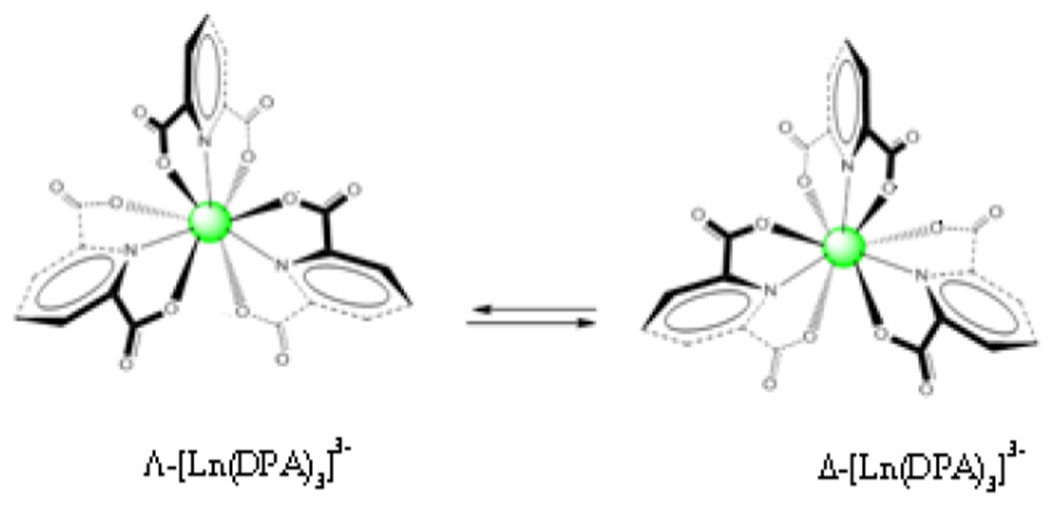
Structures of the Λ-[Ln(DPA)_3_]^3−^ **(left)** and Δ-[Ln(DPA)_3_]^3−^ **(right)** complexes showing the helical wrapping of achiral 2,6-pyridinedicarboxylate (DPA) ligands for 1:3 Ln^3+^:DPA.

The origin of chirality for [Ln(L)_3_]^3−^ complexes arises from the helical wrapping of the equivalent achiral ligands coordinated to the central lanthanide (Eu^3+^ or Tb^3+^). Depending upon the directionality of the helical twist of the ligands (right or left), the complex that is formed is labeled as Λ (left twist) or Δ (right twist). The Λ and Δ forms of the [Ln(L)_3_]^3−^ complex are in fact enantiomers, as they are non-superimposable mirror images and they exist in solution as a racemic mixture (Figure 1). The complex equilibrium, once established, can be preferentially perturbed by the addition of a chiral molecule (CEC* = chiral environment compound, * = denotes the chiral center of the chiral molecule) such as an amino acid. It is generally assumed that the perturbation caused by the CEC* occurs without any significant distortion of the local D_3_ structure of the lanthanide(III) complex. The interaction between the CEC* and the preferred form of the lanthanide(III) complex occurs through the second coordination sphere of the ligands [23–28,48–50,52–54], The following Equations (l)–(3) illustrate the preferential perturbation, described with relevant equilibria [23–28,48–50,52–54].

(1) $$\Delta -{\left[\mathrm{Ln}(\mathrm{DPA}{)}_{3}\right]}^{3-}⇌\Lambda -{\left[\mathrm{Ln}(\mathrm{DPA}{)}_{3}\right]}^{3-}(K_{\mathrm{rac}}=1)$$

(2) $$\Delta -{\left[\mathrm{Ln}(\mathrm{DPA}{)}_{3}\right]}^{3-}+{\mathrm{CEC}}^{*}⇌\Delta -{\left[\mathrm{Ln}(\mathrm{DPA}{)}_{3}\right]}^{3-}:{\mathrm{CEC}}^{*}(K_{1})$$

(3) $$\Lambda -{\left[\mathrm{Ln}(\mathrm{DPA}{)}_{3}\right]}^{3-}+{\mathrm{CEC}}^{*}⇌\Lambda -{\left[\mathrm{Ln}(\mathrm{DPA}{)}_{3}\right]}^{3-}:{\mathrm{CEC}}^{*}(K_{2})$$

Equation (1) shows the unperturbed complex equilibrium that is formed between the Λ and Δ forms of [Ln(DPA)_3_]^3−^ before a chiral CEC* is introduced. Equations (2) and (3) show the perturbation and the formation of the outer-sphere association complexes that form after the addition of the CEC*. The more abundant adduct present (denoted in Equations (2) and (3) by a colon (:)) in solution is dependent upon the stability of the association between the ligand blades of the complex and the CEC*. The degree of perturbation is commonly reported as the luminescence dissymmetry ratio, or *g*_*lum*_. The magnitude of the *g*_*lum*_ illustrates how efficiently the CEC* is able to perturb the complex equilibrium. The sign of the *g_lum_* (+ or −) is indicative of which form of the complex the CEC* has preferentially associated with.

The discriminatory interactions between the CEC* and the preferred form of the complex are governed through non-covalent intermolecular interactions, including, but not limited to, electrostatics, hydrogen bonding, hydrophobic interactions, *π*-stacking interactions, and Van-der Waals interactions [27–29]. The type of intermolecular interactions that are involved or that govern the perturbation would likely change, depending upon the structure and functional groups on the ligands of the complex and the CEC* [27–29]. They would also likely be further affected with the solvent environment as well in the same manner. The solvent environment may also be the key to offering a full description of the mechanism of the Pfeiffer effect [44–48].

**Scheme 1.**
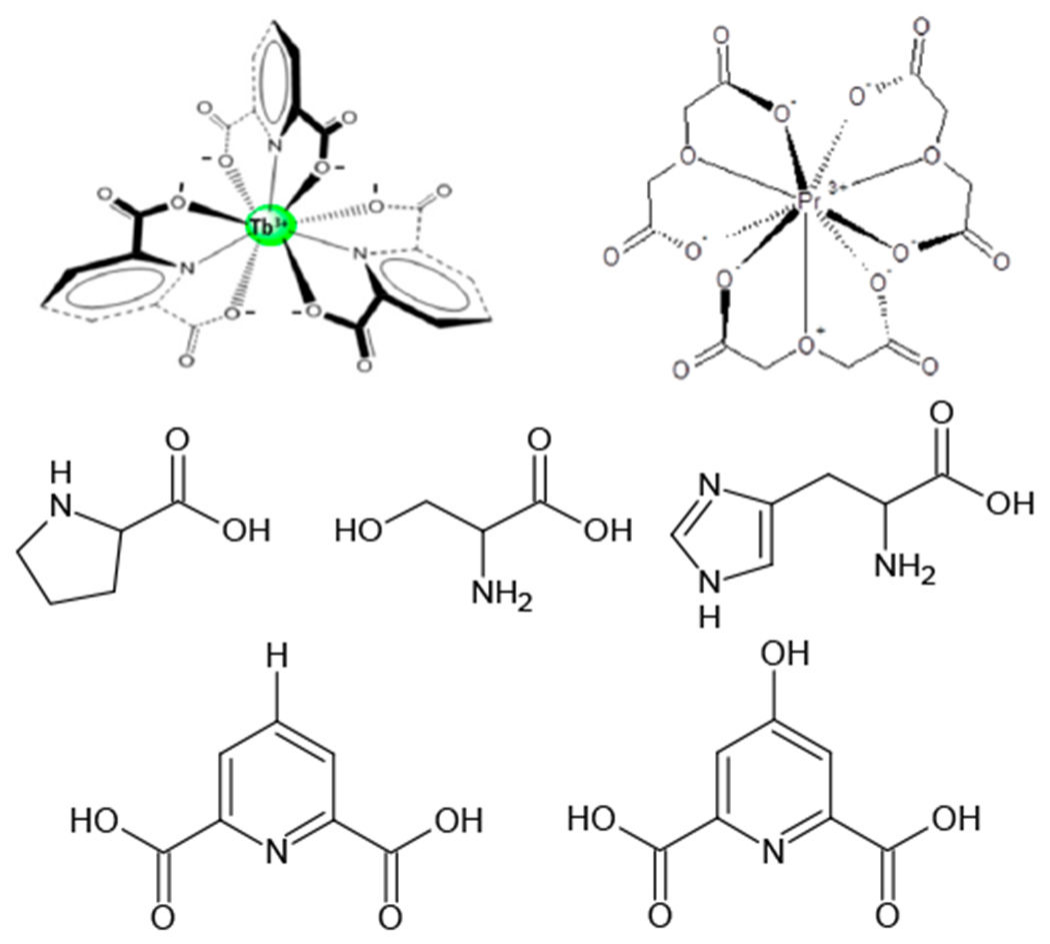
Molecular structures of [Tb(DPA)_3_]^3−^ (top left), [Pr(ODA)_3_]^3−^ (**top right**), where ODA is oxydiacetate, l-proline (**middle left**), l-serine (**middle middle**), l-histidine (**middle right**), DPA (**bottom left**), and chelidamic acid (**bottom right**).

Early attempts at describing the Pfeiffer effect include solvent studies; however, they have attributed their observations to one specific factor such as the variation of the dielectric constant of the solvent environment [48–50]. Wu et al. and Parac-Vogt et al. are just two examples of this [48–50]. Both authors utilize binary solvent systems such as formamide:water, methanol:water, and DMSO (where DMSO = dimethyl sulfoxide):water [48–50]. Wu et al. studied the Pfeiffer activity using the [Tb(DPA)_3_]^3−^ (Scheme 1) complex with the CEC* l-histidine (Scheme 1), whereas Parac-Vogt et al. studied the effect using [Pr(ODA)_3_]^3−^ (Scheme 1), where ODA is oxydiacetate [49,50].

It is also important to point out that Wu et al. used CPL spectroscopy, and Parac-Vogt et al. used CD spectroscopy, to perform their respective studies [48–50]. Both authors observed similar results when the dielectric constant of the solvent environment was either increased or decreased with an organic solvent with a dielectric constant that was either higher or lower, compared to that of water (*ε* = 80). When the binary solvent systems of formamide:water were used, both authors observed a decrease (smaller perturbation) in the | *g*_*lum*_ | or | *g*_*abs*_ | (where | *g*_*abs*_ | = absorption dissymmetry ratio) and an increase (larger perturbation) was observed when the binary solvent system of DMSO:water was used [48–50]. Wu et al. also utilized the binary solvent system consisting of methanol:water in which an increase in the | *g*_*lum*_ | was observed [48]. Had Parac-Vogt et al. also included the methanol:water system, an increase would have more than likely also been observed in the | *g*_*abs*_ | as well. Both Wu [47] and Parac-Vogt et al. [48,49] concluded that the increase in the | *g*_*lum*_ | or | *g*_*abs*_ | was contributable to the increase or decrease of the dielectric constant of the solution when an organic solvent with a higher dielectric than water (*ε* = 80) was introduced [47–49].

This was the only attributable explanation offered regarding the Pfeiffer effect mechanism. While the dielectric constant does have an effect to a certain extent on the molecular mechanism, it is not the only factor to consider when attempting to offer a more complete description of the effect. It would be more likely the case if it was an ideal solvent environment; however, since it is a binary solvent system with the lanthanide(III) complex and the amino acid present; it is less likely that the effect is exclusively attributed to just the dielectric constant. The mechanism of the Pfeiffer effect is more complex than to just descriptions of observations with a singular factor. The solvent environment itself may be the key to offering a more complete description. This work is part of a series of ongoing efforts within our laboratory to provide/offer a more detailed description of the mechanism of the Pfeiffer effect. We also hope to utilize the information gained from this series to better predict future CPL studies that are based on the chiral recognition of amino acids and other molecules with biological significance.

## 2. Results and Discussion

### 2.1. Solvent Study

In this work, we have chosen to study the 9-coordinate Tb(III) and Eu(III) complexes with the achiral ligand DPA. This system was specifically chosen because there are a number of studies for where this particular complex is involved [23–26,52,53]. In addition, there is also much known regarding the CPL activity of the [Tb(DPA)_3_]^3−^ complex, and as well as its stability and luminescence properties [23–26,52]. The DPA (scheme 1) ligand is also structurally simplistic, as the aromatic ring is devoid of any other functional groups except hydrogen atoms. The absence of complicated functional groups allows the factoring out of any possible interference from the interaction of the CEC* (l-serine or l-proline, Scheme 1) with a functional group on the DPA ligand. l-serine and l-proline were also chosen as the CEC*s, due to numerous unpublished studies that were conducted within our group. They were also chosen due to their individual structural characteristics. l-serine possessed a freely rotating carboxylate group, whereas l-proline possessed a carboxylate group that was constricted from movement. We wanted to explore the effects of a freely rotating carboxylate versus a restricted carboxylate, as we know that the point of interaction is with the positively charged amino group on both l-serine and l-proline.

Take the DPA derivative chelidamic acid (CDA, Scheme 1) which had a hydroxyl group grafted to the 4-position of the pyridine ring. A majority of the interactions observed between the CDA (chelidamic acid) ligands are attributed to the interaction of the hydroxyl function with the CEC* [28]. The absence of functional groups on the DPA ring may also aid in the identification of specific solvent interactions, such as specific solvent packing effects, and perhaps certain chiral imprinting effects on the solvent sheaths by the complex and the CEC* [54–59].

The solvents were chosen in accordance with previous authors and similar solvents were added in order to expand the list and range of dielectric constant [48–50,54]. The dielectric constant of each solvent is listed in Table 1 from smallest to largest. Additional solvents were also added to the list based upon their structural characteristics and on what type of intermolecular interactions they could possibly form with the solvent environment, DPA ligand, and the CEC*.

**Scheme 2.**
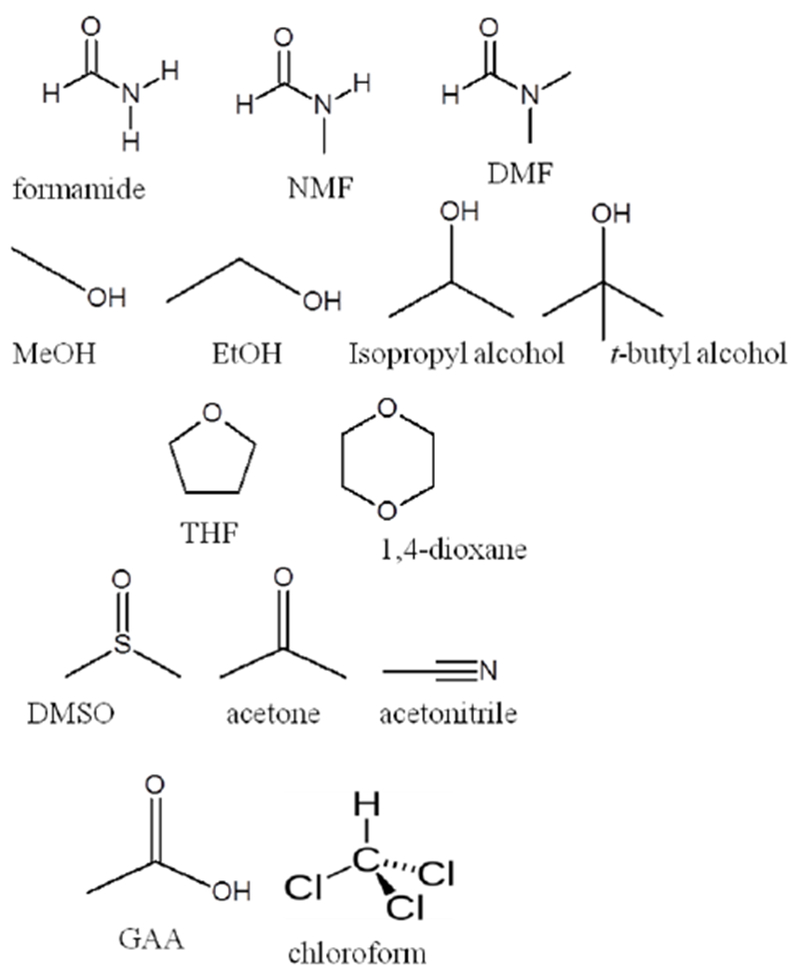
Molecular structures of the various solvent families utilized in this work.

The molecular structures of the solvents are shown in Scheme 2. Each solvent was later arranged into a particular family according to similarities in functional groups (Scheme 2). All data reported are for systems that contain 0.005 M [Tb(DPA)_3_]^3−^ with 40 equiv. (where equiv. = equivalents) of l-serine. The concentration of [Tb(DPA)_3_]^3−^ and the equiv. of l-serine were held constant throughout each experiment. The pH was also maintained within a range that was equivalent to physiological pH (6–7). This was also the pH range at which the amino acid l-serine is in its zwitterionic form, and the D_3_-[Tb(DPA)_3_]^3−^ complex equilibrium is established [23–33,35]. The Eu(III)-based complex ([Eu(DPA)_3_]^3−^) was also used with the same parameters, as mentioned for the [Tb(DPA)_3_]^3−^ experiments; however, only the formamide solvents were used with this system (see explanation later).

### 2.2. The Formamide Solvents

**Figure 2.**
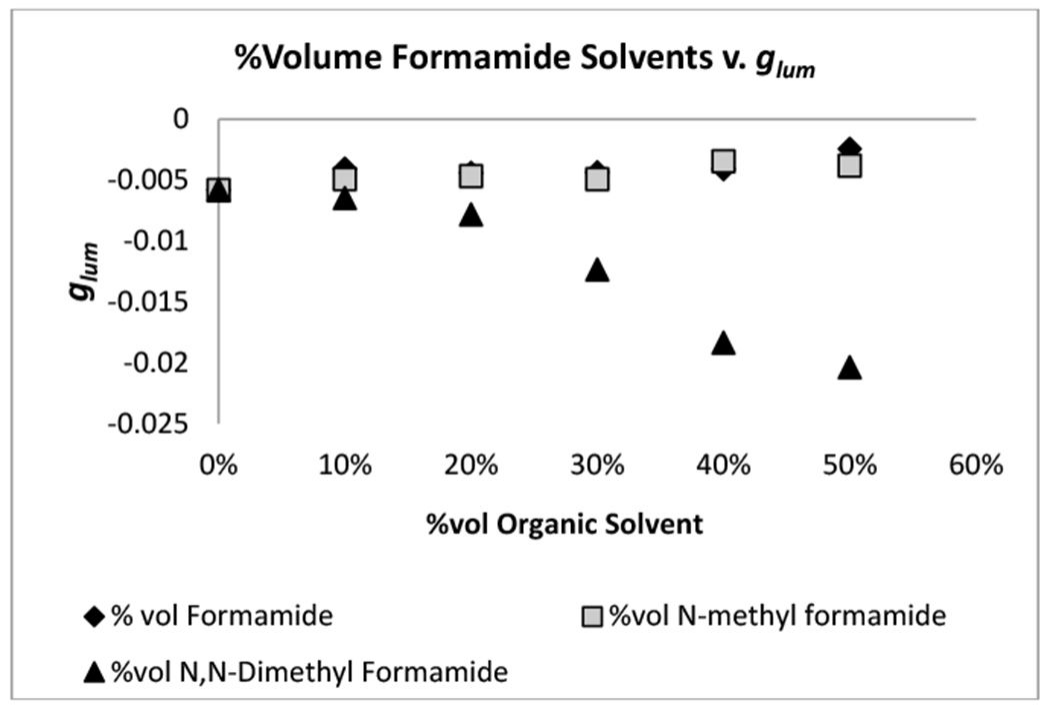
*g*_*lum*_[Tb(DPA)_3_]^3−^ with formamide (circle), *N*-methyl formamide (square), and *N*,*N*-dimethyl formamide (triangle).

The formamide solvents presented results that were unlike those observed for the other solvent families (Figure 2, Table 2). We saw a significant decrease in the | *g*_*lum*_ | with formamide and *N*-methyl formamide but a significant increase in the | *g*_*lum*_ | for *N*,*N*-dimethyl formamide was observed (Figure 2, Table 2). In the case of the other solvent families, a general increase was observed in the | *g*_*lum*_ | for all families of solvents except the outliers, glacial acetic acid and chloroform. According to both Nguyen et al. and Moussa et al., the point of association of the amino acid with the ligand blades of the complex occurs through the nitrogen atoms on the amino acid [27,28]. Assuming that this is the case with every amino acid or CEC* bearing a nitrogen atom, it was assumed that the same might be occurring with the formamide solvents as well, since they also have nitrogen atoms present. In addition, it was also proposed that since formamide and *N*-methyl formamide both have exposed nitrogen atoms, they might be outcompeting the amino acid (l-serine) for an association to the complex. Another possibility was that the two formamide solvents replaced a DPA ligand, which could have also accounted for the significant drop in the | *g*_*lum*_ |. In order to confirm or refute either of the proposed possibilities ^5^D_0_ ← ^7^F_0_ Eu(III) excitation was done in order to determine the speciation of the Eu(III)-containing complex. Due to the theoretically simpler crystal field energy level pattern of Eu(III), it was possible to determine the unique environment of the Eu(III) ion in solution. If more than one species of Eu(III) complex was present it would be apparent in the resulting excitation spectra as either a second distinct peak or a shoulder peak at or near the maxima [28]. The fact that a | *g*_*lum*_ | was still observed with the [Tb(DPA)_3_]^3−^ system, and when formamide or *N*-methyl formamide (Figure 2, Table 2) was used as the secondary solvent relative to water, suggested that the solvents did not replace a ligand on the Eu(III) complex. This conclusion was made based on the fact that only one peak was observed in the resulting Eu(III) excitation spectra for each formamide solvent.

**Figure 3.**
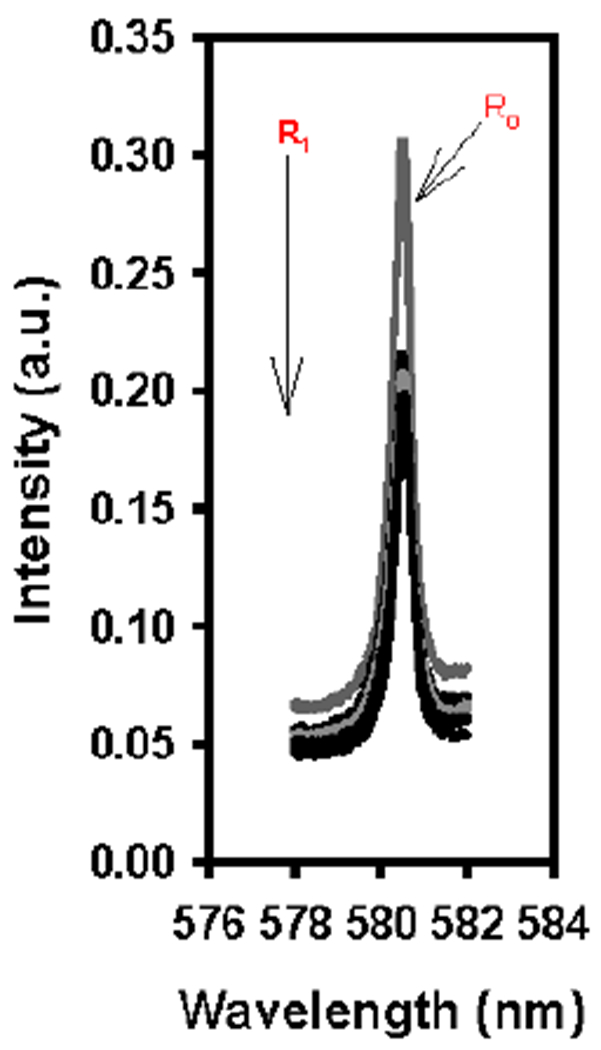
R_1_: ^5^D_0_ ← ^7^F_0_ (Eu) excitation spectra for solutions containing 0.005 M [Eu(DPA)_3_]^3−^, 40 equiv. l-serine with solvent ratios (H_2_O:formamide) from 90:10 to 50:50. R_0_: ^5^D_0_ ← ^7^F_0_ (Eu) excitation spectra for the control containing 0.005 M [Eu(DPA)_3_]^3−^, 0 equiv. l-serine, and 50:50 H_2_O:formamide.

**Figure 4.**
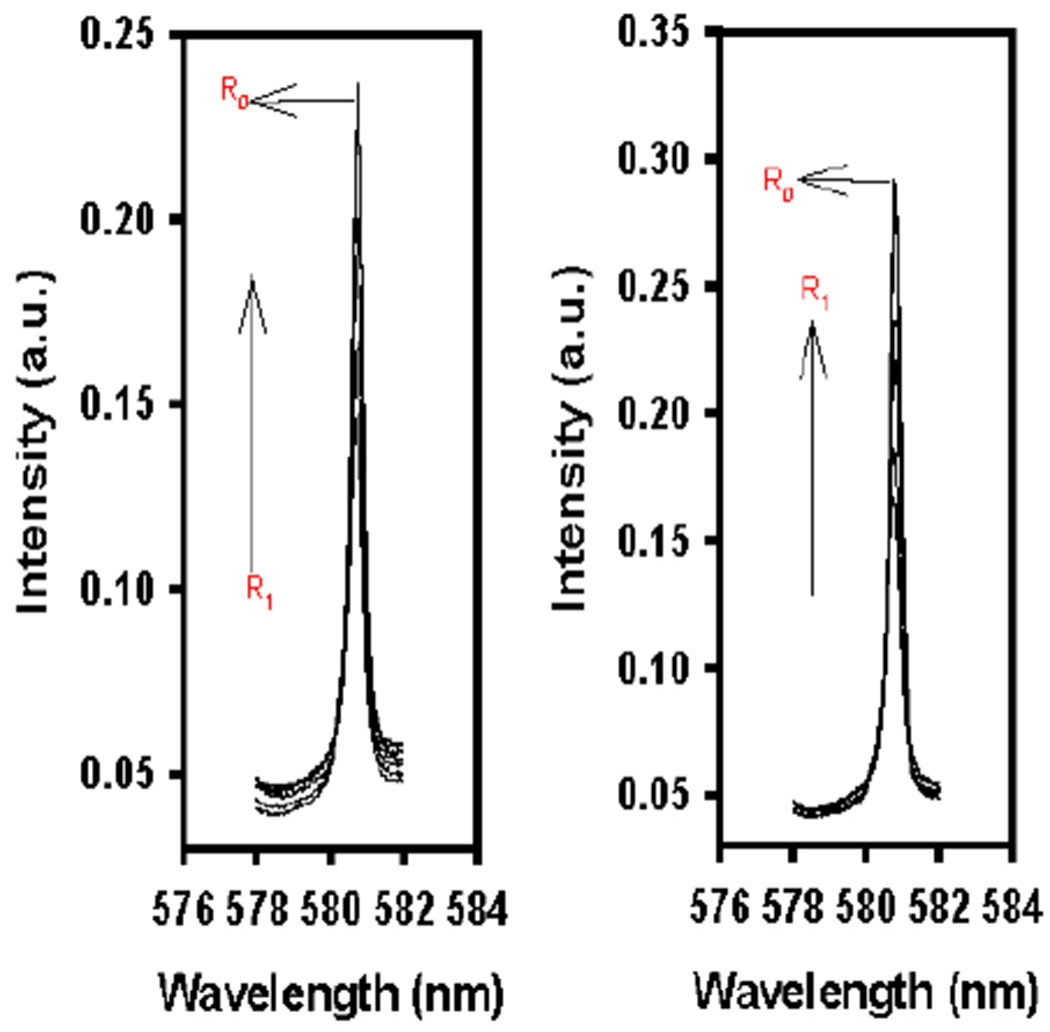
R_1_: ^5^D_0_ ← ^7^F_0_ (Eu) excitation spectra for solutions containing 0.005 M [Eu(DPA)_3_]^3−^, 40 equiv. l-serine with solvent ratios of H_2_O:NMF (left) and H_2_O:DMF (right) from 90:10 to 50:50. R_0_: ^5^D_0_ ← ^7^F_0_ (Eu) excitation spectra for the control containing 0.005 M [Eu(DPA)_3_]^3−^, 0 equiv. l-serine, and 50:50 H_2_O:NMF (**left**) and H_2_O:DMF (**right**), respectively.

^5^D_0_ ← ^7^F_0_ (Eu) excitation spectroscopy was also done for that of solutions with *N*,*N*-dimethyl formamide for a comparison to Eu(III)-excitation for formamide and *N*-methyl formamide. Solutions were prepared containing 0.005 M [Eu(DPA)_3_]^3−^ 40 equiv. l-serine with either formamide, *N*-methyl formamide, or *N*,*N*-dimethyl formamide in ratios (H_2_O:solvent) of 90:10, 80:20, 70:30, 60:40, and 50:50. A solution containing the 0.005 M [Eu(DPA)_3_]^3−^ and a 50:50 ratio H_2_O:solvent was also prepared, excluding the 40 equiv. l-serine, which was used as a control. The results for the formamide are shown in Figure 3, and the results for *N*-methyl formamide and *N*,*N*-dimethyl formamide are shown in Figure 4.

Here at all ratios, only one peak is observed (maximum being centered around 580–581 nm, (Figure 3)) which is representative of only the 1:3 species of [Eu(DPA)_3_]^3−^ being present in the solution. The conclusion is made, then, that the formamide solvent did not replace the ligand on the complex. It is however, a possibility that formamide is still competing with l-serine for association to the complex.

Since both l-serine and formamide have NH groups present, they are capable of forming hydrogen bonding interactions, which we know from previous studies are important for the CEC to interact with the complex and to induce the perturbation of the racemic complex equilibrium [27,28]. While there may be some competition between formamide and l-serine, there is still a perturbation occurring (Table 2). Since perturbation was still observed when formamide was introduced as the secondary solvent, and only a single peak was observed in the ^5^D_0_ ← ^7^F_0_ (Eu) excitation spectra (Figure 3), it can be theorized that the amino acid and the formamide solvent still had some influence over the perturbation of the racemic complex equilibrium. However, it was not a significant perturbation (*g*_*lum*_ = −0.00406, −0.00440, −0.00429, −0.00408, and −0.00242 at ratios of 90:10, 80:20, 70:30, 60:40, and 50:50 H_2_0:formamide, respectively). With that, there was a possibility that something more significant than simple associative competition occurred between the formamide solvent and l-serine. Another possible explanation is that the amino groups of l-serine and formamide formed strong hydrogen bonds with each other. If this is the case, formamide would be hindered from forming hydrogen bonding interactions with the water molecules that were thought to be present within the solvation sheath of the complex itself.

The results for the ^5^D_0_ ← ^7^F_0_ (Eu) excitation experiments where *N*-methyl formamide or *N*,*N*-dimethyl formamide were used as the secondary solvent, are shown in Figure 4. Comparing the 50:50 (H_2_O:solvent) peaks for *N*-methyl formamide (left) and 50:50 (H_2_O:solvent) *N*,*N*-dimethyl formamide (right), to the 50:50 (H_2_O:solvent) peak for that of formamide (Figure 3), there was a clear difference in intensity between all three solvents at the 50:50 ratio (R_1_), the peak for *N*,*N*-dimethyl formamide being the most intense. The main difference between the three formamide solvents is the presence or lack of methyl groups on the nitrogen (Figures 3 and 4). As has already been proposed, formamide may be hydrogen bonding with l-serine, and the aldehyde hydrogen is left free to interact with water molecules within the solvation sheath of the complex. The hydrogen may not be a large enough or hydrophobic enough group to influence the necessary movement/rearrangement of the water molecules in the solvation sheath. As a consequence of this, the complex equilibrium appears to only experience a minimal shift, which is evident in the | *g*_*lum*_ | data (Table 2). Comparing the excitation spectra for formamide, *N*-methyl formamide, and *N*,*N*-dimethyl formamide, it seems more likely that hydrophobic groups on secondary solvents were necessary in order to influence the perturbation of the complex equilibrium. The data collected from the following experiments seem to also support the theory that hydrophobic groups are necessary to influence the perturbation of the complex equilibrium.

### 2.3. Alcohol Solvents

**Figure 5.**
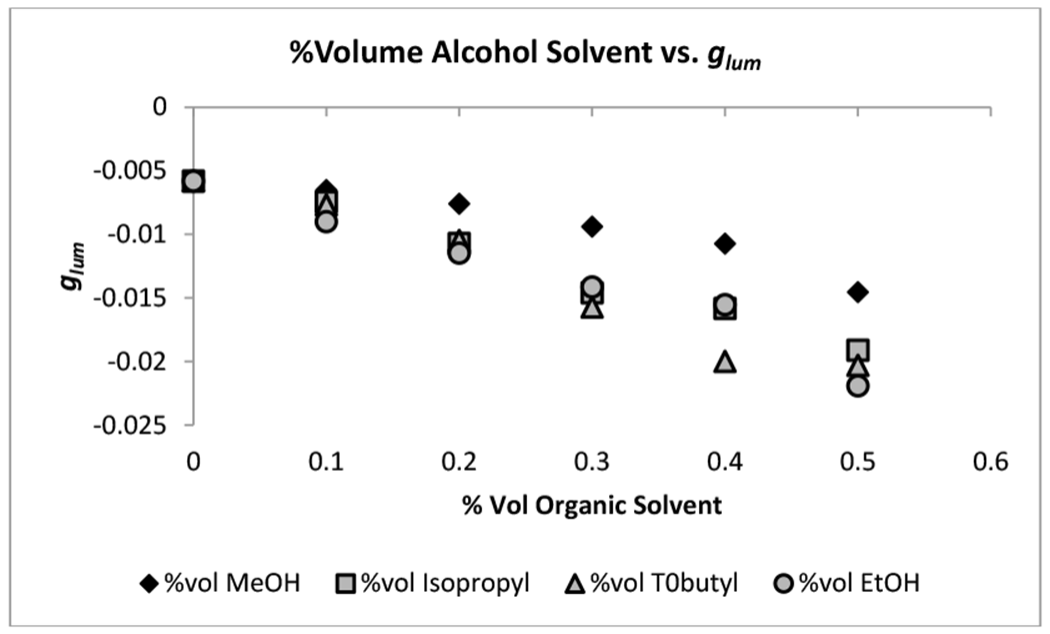
Plots of luminescence dissymmetry values (*g*_*lum*_) of [Tb(DPA)_3_]^3−^ with varying ratios of either methanol (diamonds), ethanol (circle), isopropyl alcohol (square), or *t*-butyl alcohol (triangle). Ratios are varied from 90:10 to 50:50 H_2_O:alcohol solvent.

The [Tb(DPA)_3_]^3−^ complex was used in place of [Fu(DPA)_3_]^3−^ for the following and remaining experiments. Each alcohol was varied in ratios of H_2_O:alcohol from 90:10 to 50:50 with 0.005 M [Tb(DPA)_3_]^3−^ and 40 equiv. of l-serine. The pH of each series of solutions was held at or near physiological pH (6–7 pH). The effect of adding alcohols as the secondary solvent to an l-serine perturbed [Tb(DPA)_3_]^3−^ system is shown in Figure 5 and Table 3. Each of the alcohols (methanol, ethanol, *t*-butyl alcohol and isopropyl alcohol) contained hydrophobic side chains and caused the | *g*_*lum*_ | to become more negative (Figure 5, Table 3). The large negative value of the *g*_*lum*_ was suggestive that either the Λ or the Δ form of the complex was in greater abundance over the other form. This observation was consistent with all of the experiments reported here, with the exception of formamide and *N*-methyl formamide, in which less of a perturbation (smaller | *g*_*lum*_ |) was observed for formamide (Figure 2, Table 2). If it is assumed that the amino group of l-serine is hydrogen bonded to the hydroxyl hydrogen of the alcohol solvents, it would be plausible to assume that the hydrophobic substituents of the alcohols would be oriented toward the solvation sheath of the [Tb(DPA)_3_]^3−^ complex. This orientation would force the necessary rearrangement of the solvation sheath. The rearrangement that occurs between the water molecules in the solvation sheath of the complex and the hydrophobic groups of the organic solvents may be very similar to that of water molecule exclusion from hydrophobic regions of proteins [44,45].

We also was evidence that hydrophobic substituents had an influence on the perturbation in the formamide and derivative ^5^D_0_ ← ^7^F_0_ (Eu) excitation experiments as well, specifically when the concentration of *N*,*N*-dimethyl formamide is increased (Figure 4, Table 2). It was also observed with *N*-methyl formamide at higher concentrations (Figure 4, Table 2). If the hydrocarbon chains of the alcohol solvents did in fact have this effect, then smaller chain alcohols such as methanol would not have such a large effect compared to the other alcohols with larger hydrophobic side chains, as was apparently first proposed in the work of Schipper [54], The | *g*_*lum*_ | data from Table 3 indicate that methanol does in fact have less of an effect on the perturbation than the other alcohols at each ratio. The effect then would likely become greater as the branching of CH groups on the alcohol increases, but it may taper off when the concentration of bulkier alcohol increases, due to the introduction of unfavorable steric interactions between bulky hydrocarbon chains.

From the | *g*_*lum*_ | behavior alone with the alcohol solvents and the formamide solvents it seems that hydrocarbon groups combined with the presence of groups capable of hydrogen bonding with the CEC* are important in order to influence a significant perturbation. The dielectric constant as proposed by previous authors [48–50] seems to be less important, but it cannot be ruled out completely. The remaining solvents in this study seemed to add validity to this as well.

### 2.4. Tetrahydrofuran and 1,4-Dioxane

**Figure 6.**
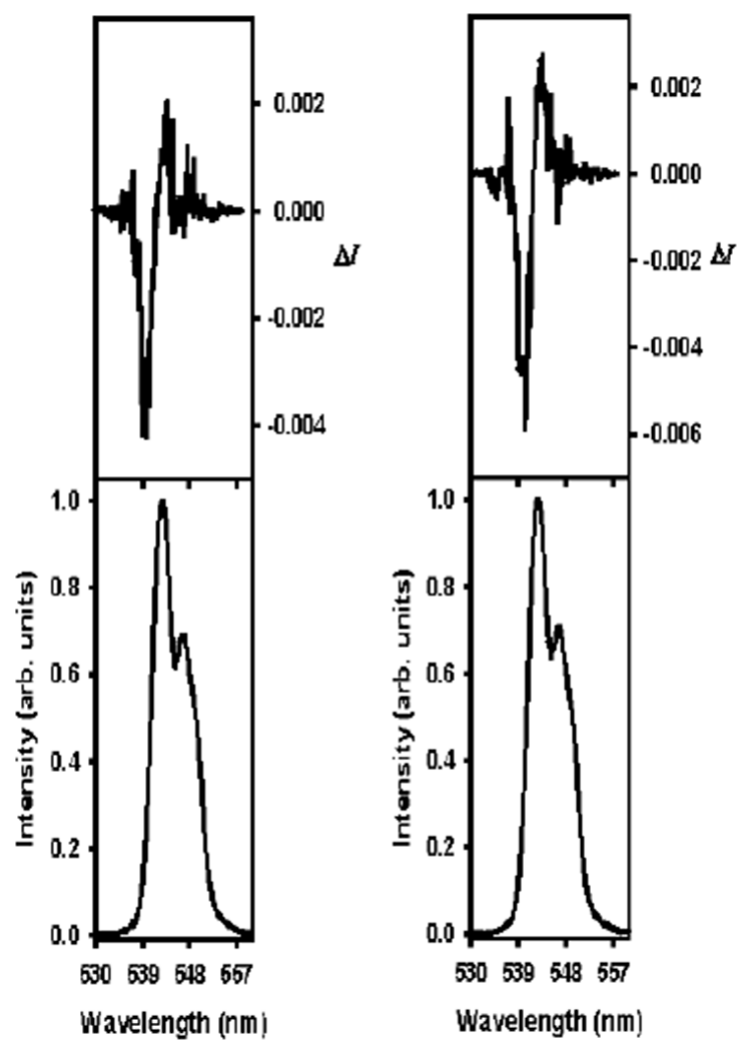
Circularly polarized luminescence (CPL) (top curves) and luminescence (lower curves) spectra for the ^5^D_4_ → ^7^F_5_ transition of [Tb(DPA)_3_]^3−^ with 40 equiv. l-serine for 100:0 H_2_O (**left**) and 90:10 H_2_0:THF (**right**).

Tetrahydrofuran and 1,4-dioxane were chosen because they have hydrogen carbon ring structures and they are both hydrogen bond acceptors with relatively low dielectric constants compared to water [60]. As Table 4 shows, all of the solvents in this group caused a significant perturbation when compared to the perturbation with water as the only solvent (−0.00581) at all of the ratios examined. It seems that from the observations with the formamides and the alcohols, the assumption that hydrophobic groups are essential in the mechanism of perturbation are more likely to be the case. Hydrophobic groups are essential as they may force the water molecules in the solvation sheath of the complex to pack closer to the central Tb^3+^, which enhances the chiral structure of the complex itself [54]. Since 1,4-dioxane is a bulkier solvent than tetrahydrofuran, it should have a greater effect on the perturbation than the less bulky tetrahydrofuran molecule; the | *g*_*lum*_ | data seemed to support this as well. This is also consistent with what was observed with ethanol and the bulkier alcohol solvents. In order to further investigate the influence of the hydrocarbon rings and to validate that these groups have an effect on the behavior of the water molecules in the solvation sheath of the complex, CPL spectra were run at each ratio with the solvent that showed the greatest perturbation (largest | *g*_*lum*_ | value). CPL was also run on a sample with 0.005 M [Tb(DPA)_3_]^3−^, 40 equiv. of l-serine and water as the only solvent to act as a control (Figure 6, top left). The 60:40 ratio was not included as it was originally prepared for a sample, where acetone was used as the secondary solvent. Acetone was originally included in this group, but it was later regrouped with the polar aprotic solvents, as acetone was more structurally similar to the polar aprotic solvents than with tetrahydrofuran and 1,4-dioxane. Considering the | *g*_*lum*_ | value at the 60:40 ratio for 1,4-dioxane, it was assumed that the CPL spectrum for this ratio with 1,4-dioxane would be similar to the CPL for the 50:50 ratio, with 1,4-dioxane as the secondary solvent.

**Figure 7.**
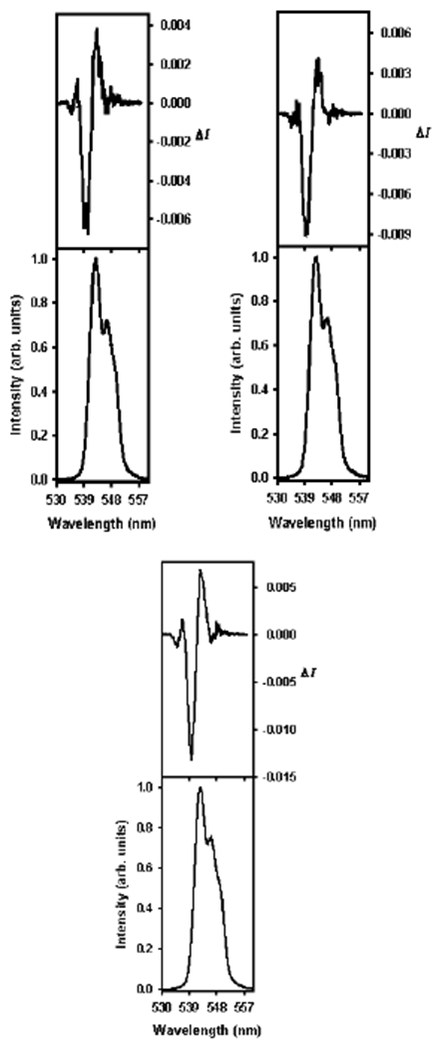
CPL (top curves) and luminescence (lower curves) spectra for the ^5^D_4_ → ^7^F_5_ transition of [Tb(DPA)_3_]^3−^ with 40 equiv. l-serine for 80:20 H_2_0:l,4-dioxane (**top left**), 70:30 H_2_0:l,4-dioxane (**top right**), and 50:50 H_2_0:l,4-dioxane (**bottom**).

**Figure 8.**
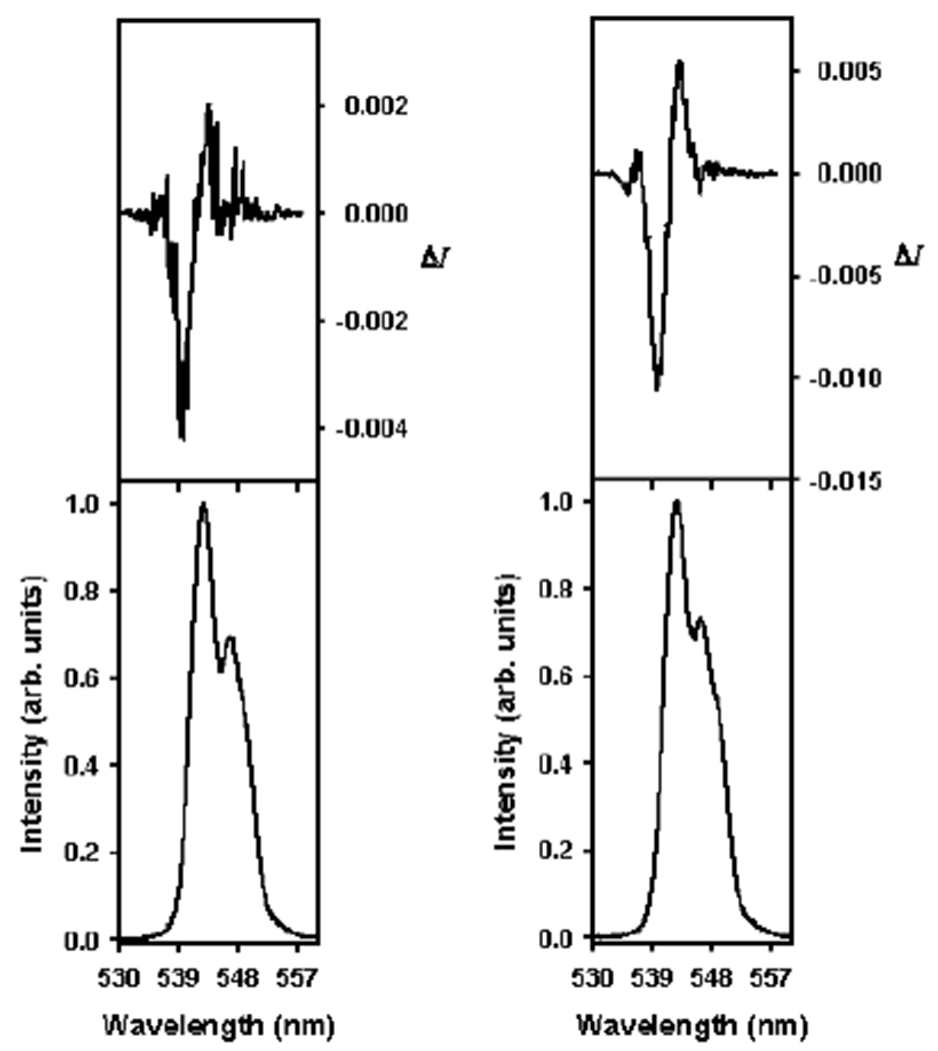
CPL (top curves) and luminescence (lower curves) spectra for the ^5^D_4_ → ^7^F_5_ transition of [Tb(DPA)_3_]^3−^ with 40 equiv. l-serine for 100:0 H_2_:solvent (**left**) and 60:40 H_2_O:acetone (**right**).

Figure 6 shows the results of the CPL experiment with the 100:0 H_2_O:solvent control and the 90:10 H_2_O:tetrahydrofuran ratio. The results of CPL experiments with H_2_O:1,4-dioxane ratios of 80:20, 70:30, and 50:50 are shown in Figure 7. It should be pointed out that the CPL spectra were obtained through the excitation of the DPA ligands of the [Tb(DPA)_3_]^3−^ complex, rather than the direct excitation of the lanthanide(III), as in ^5^D_0_ ← ^7^F_0_ (Eu) excitation spectroscopy. Examining the CPL spectra (Figures 7 and 8) of the l-serine perturbed [Tb(DPA)_3_]^3−^ sample, with only water as the solvent, with the CPL of a similar sample with 1,4-dioxane as the secondary solvent, the spectra were more defined, as the percentage of 1,4-dioxane increased. It is likely that the hydrophobic ring structure of 1,4-dioxane (Scheme 2) caused the water molecules in the solvation sheath of the [Tb(DPA)_3_]^3−^ complex to pack tighter around the DPA ligand blades which forced the ligands into closer proximity to the lanthanide(III) at the center of the complex. As a direct consequence of this, the chiral structure of the complex becomes more defined, this could be what the CPL spectra for 1,4-dioxane samples are showing (Figure 7, Table 4). It becomes increasingly more likely when the CPL spectra of samples with 1,4-dioxane are compared to the CPL spectra with tetrahydrofuran as the secondary solvent. There is a clear increase in the definition of the CPL curve when 1,4-dioxane is the secondary solvent than with tetrahydrofuran. Since tetrahydrofuran is less bulky (smaller ring structure) than 1,4-dioxane it should have less of an effect at higher percentages, and more of an effect at lower percentages, similar to that which has been described for ethanol. It would be advantageous for future studies to include the CPL of an entire ratio (90:10 to 50:50) series of solvent families, such as those mentioned in this work.

At this early juncture, it may be plausible to conclude that hydrophobic interactions between solvent molecules and the solvation sheath of the [Ln(DPA)_3_]^3−^ complex are indeed important to the efficient perturbation of the complex equilibrium, and that they may offer a more detailed description of the Pfeiffer effect.

### 2.5. Polar Aprotic Solvents

The solvents in this family were chosen as they had no structural similarities to the other solvents in this work. Acetone however, as mentioned previously, was originally included with 1,4-dioxane and tetrahydrofuran. It was later grouped here, as it shared certain structural characteristics with dimethyl sulfoxide (DMSO) and acetonitrile. The three solvents, DMSO, acetonitrile, and acetone, are all considered to be polar aprotic solvents, the strength of which was determined by the dielectric constant for the solvent (Table 1) [61]. From the | *g*_*lum*_ | data shown in Table 5, acetone is the solvent, with which the perturbation is greatest in the higher ratios of 70:30 (−0.01616), 60:40 (−0.02274), and 50:50 (−0.02697).

At lower ratios (90:10 and 80:20), acetonitrile and DMSO influence the greater perturbation of the complex equilibrium (90:10 H_2_O:acetonitrile, −0.00926, and 80:20 H_2_O:DMSO, −0.01263). At first inspection, it was apparent that there was again no direct correlation between the solvent dielectric (Table 1) and the perturbation of the complex equilibrium, as the dielectric of the solution was either increased or decreased by the addition of a solvent with a larger or smaller dielectric constant than water (80). The only correlation between the dielectric of the solvent and the perturbation of the complex equilibrium was only observed at the 70:30 ratio for all the solvents studied. Since it is not the dielectric constant (increasing or decreasing) of the solvent that is responsible for the perturbation, it must again be attributable to the solvent structures (Scheme 2).

It is possible that the most significant difference between the solvents in this group was the strength of their respective hydrogen bond acceptor strengths. DMSO is classified as a stronger hydrogen bond donor compared to both acetone and acetonitrile [61]. This is due to the presence of the sulfur atom, which is much more polarizable than carbon and less electronegative than oxygen or nitrogen. If the acceptor strength of the solvent was indeed the main influence, DMSO should have had the greatest influence on the perturbation in the higher ratios (Table 5), but acetone had the greater perturbation at the higher ratios. DMSO only perturbed the complex equilibrium better than acetone and acetonitrile at the 80:20 ratio (−0.01263) but the difference between the | *g*_*lum*_ | value for DMSO and acetone was a significant difference, so the acceptor strength cannot be completely discarded. It has an effect to some extent, but that effect remains to be discovered.

It was apparent from the CPL spectrum for acetone (Figure 8) that for a CPL for a perturbed solution of [Tb(DPA)_3_]^3−^ and a solvent ratio of 60:40 H_2_O:acetone, the spectrum was more defined than the corresponding system, with water as the only solvent. The increasing definition or shape of the CPL signal could be attributed to the stabilization of the crystal field in the CPL spectra for 1,4-dioxane and tetrahydrofuran. It may be that the structures of the solvents are important to consider as well in regard to their size. Considering factors such as the size, structural characteristics, and physical properties, such as the dielectric constant, are important when attempting to study solvent effects in particular on the Pfeiffer effect mechanism.

It seems that the presence of the hydrophobic groups on these solvents had the same effect as those described with the alcohol solvents and as well as with DMF, and 1,4-dioxane, and THF. It has been proposed in previous sections in this work and as well as for the aprotic solvents, that hydrophobic substituents are indeed important, in order to force the rearrangement of the water molecules in the solvation sheath of the [Tb(DPA)_3_]^3−^ complex. The rearrangement of the solvation sheath of the complex is essential as it enhances the chiral structure of the complex. It may also introduce a second source of chirality in the solvation sheath itself via chirality transfer [3,20–22,54–58,62–65]. If the water molecules are packed around the ligand blades in such a manner that the solvation sheath retains the shape of the complex, the chirality transfer from the complex to the solvation sheath is more efficient [3,20–22,54–58,62–65]. This transfer [55–58,62–65] may be an important aspect to consider as well in future experiments.

### 2.6. The Outliers: Glacial Acetic Acid and Chloroform

Glacial acetic acid (GAA) and chloroform were added as outliers. Glacial acetic acid was added because Schipper [53] mentioned that they observed a sign change in the | *g*_*lum*_ | value when glacial acetic acid was used as the secondary solvent [53]. It is also important to point out that Schipper concluded that the sign change that was observed, when GAA was added as the secondary solvent, indicated that the solvent sheaths of complexes involved in the Pfeiffer effect was important to the mechanism and warrant further investigation [53]. Glacial acetic acid was added in part because of the theory proposed by Schipper [53] and because if a sign change was observed here, it would further the theory that the solvation sheath of the complex and its rearrangement are essential to the functioning of the Pfeiffer effect. It may also be possible to add validity to the notion of a transfer of chirality from the complex to the solvation sheath. A sign change in the *g*_*lum*_ value (− to +) indicates that the form of the complex is changing. If we were also able to observe a sign change as well, using a different Pfeiffer system ([Tb(DPA)_3_]^3−^:l-serine) than Schipper [53]. ([Zn(phen)_3_]^2+^ and [Ni(phen)_3_]^2+^:*d*-bromocamphorsulfonate and *d*-cinchoninium) with the same solvent (GAA), then it can be concluded that the solvation sheaths are in fact involved, and are an extremely important factor in the mechanism of the Pfeiffer effect [53].

**Figure 9.**
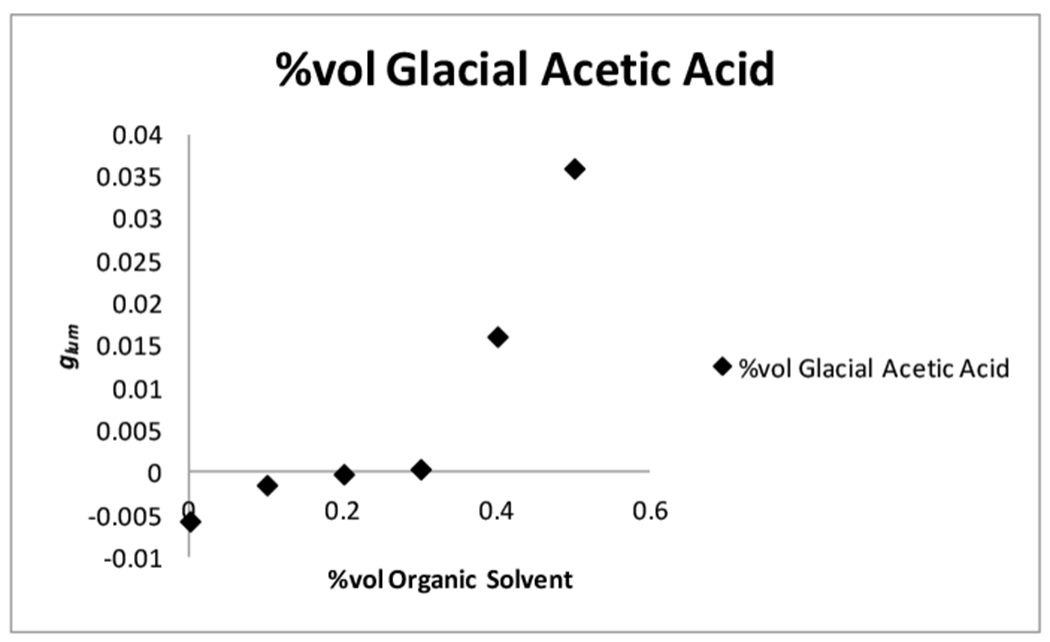
Plots of luminescence dissymmetry values (*g*_*lum*_) of [Tb(DPA)_3_]^3−^. Conditions: 0.005 M [Tb(DPA)_3_]^3−^ with 40 equiv. l-serine. Ratios of 90:10–50:50 H_2_0:GAA (where GAA = Glacial Acetic Acid), monitored at the spectral range of ^5^D_4_ → ^7^F_5_ at room temperature and pH 7.00, and measured at λ_ex_ = 283.00–289.00 nm and λ_em_ = 542.80–543.00 nm.

The results of the *g*_*lum*_ experiment on a solution of perturbed 0.005 M [Tb(DPA)_3_]^3−^ with 40 equiv. of l-serine and varying H_2_O:GAA ratios, 90:10 to 50:50 H_2_O:GAA are shown in Figure 9. Initially, there was a slight perturbation in the complex equilibrium at 10% (90:10 H_2_O:GAA) and the sign of the | *g*_*lum*_ | value is still negative. However, as the concentration of GAA was increased there was an evident sign change (− to +) indicating that the form of the lanthanide(III) complex involved in the perturbation is changing. This is consistent with the observations of Schipper [53], who also observed a sign change when using GAA as a secondary solvent relative to water [53]. The largest perturbation (+0.03597) occurs when the concentration of GAA is the highest.

The fact that a sign change was observed here and in the case of Schipper [53] with transition metal complexes is further evidence that the Pfeiffer mechanism is governed by the solvent environment, and that the CEC* (l-serine) is interacting with the solvation sheath of the complex, and not directly with the ligands of the complex.

**Figure 10.**
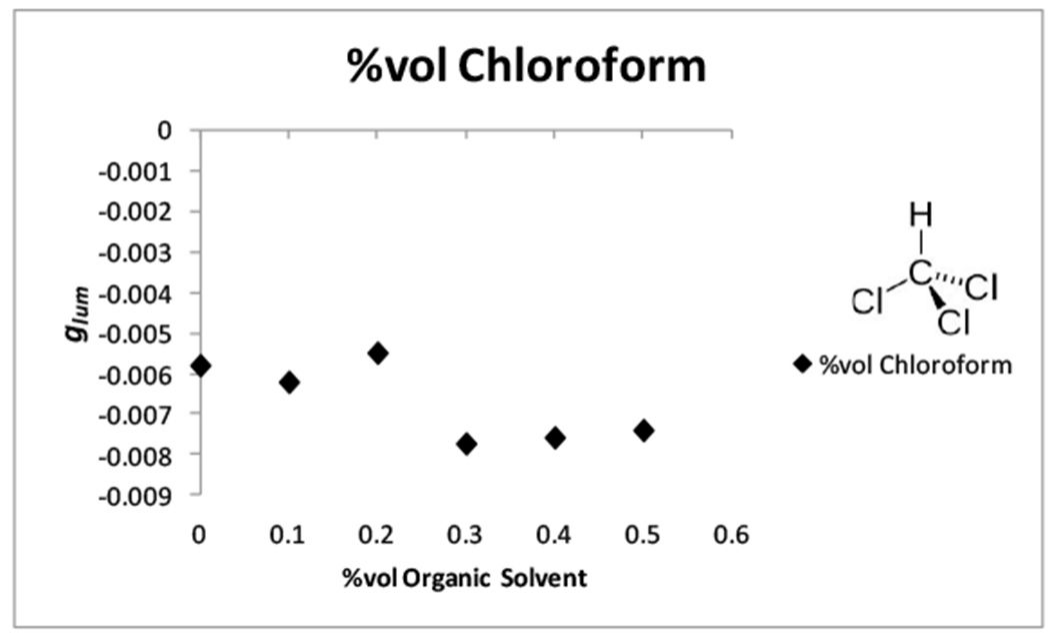
Plots of luminescence dissymmetry values (*g*_*lum*_) of [Tb(DPA)_3_]^3−^ Conditions: 0.005 M [Tb(DPA)_3_]^3−^ with 40 equiv. l-serine. Ratios of 90:10–50:50 H_2_O:chloroform, monitored at the spectral range of ^5^D_4_ → ^7^F_5_ at room temperature and pH 7.00, and measured at λ_ex_ = 283.00–289.00 nm and λ_em_ = 542.8.0–543.00 nm.

It should also be mentioned that GAA (Table 6, Figure 9) was the only solvent in the study where a sign change was observed. The reason for this may be due to the strong hydrogen bonding capability of GAA, and the fact that it can act as both a hydrogen bond acceptor through the carbonyl oxygen, or a hydrogen bond donor through the hydroxyl OH group. How exactly it interacts with l-serine and forces the contraction of the solvation sheath, is unclear from the data provided. Further investigation of GAA was not possible, as precipitates formed in the additional samples that were similarly prepared. Chloroform was an additional outlier, which was included in order to see what effect, if any, an immiscible hydrophobic solvent would have on the perturbation of the complex equilibrium (Figure 10). From the *g*_*lum*_ data collected, a plot of the *g*_*lum*_ (*y*-axis) vs. the % chloroform (*x*-axis) in solution relative to water was prepared to determine if there was an observable trend. It seems that there is no correlation between the | *g*_*lum*_ | and the percentage of chloroform in the solution. The value of the | *g*_*lum*_ |, however, remained more or less constant throughout the experiment (Table 7), which indicates that there was a perturbation occurring, but not a significant one. It must be pointed out that two layers resulted when chloroform was added to the solution, which was expected as it is an immiscible hydrophobic solvent. The water layer was chosen to run in the *g*_*lum*_ experiment because it was assumed that the complex and the amino acid would not be soluble in the chloroform layer. The chloroform layer was also tested, but no measurable *g*_*lum*_ was observed in this layer, so the conclusion was made that the perturbed complex remained in the water layer.

To further conclude that the complex was not present in the chloroform layer, the combined solution was held up to a UV lamp. The characteristic green color of Tb(III) luminescence was only seen in the water layer, and not in the chloroform layer. It is interesting that at higher chloroform ratios, the perturbation of the complex equilibrium was better than when compared to a perturbed solution with water as the only solvent. This could be an indication that the presence of chloroform had a small effect on the rearrangement of the solvation sheath of the complex through hydrophobic interactions with water molecules in the bulk solvent. This could force water molecules in the solvation sheath of the complex to pack tighter around the ligand blade of the complex. Since chloroform is immiscible with water, it is logical to conclude that the presence of chloroform has the same effect on the solvation sheath of the complex that miscible solvents with hydrophobic groups have on the solvation sheath. The effect was smaller in the presence of chloroform because it occurred through the bulk solvent instead of the primary solvation sheath of the complex.

### 2.7. Influence of Ionic Strength

**Figure 11.**
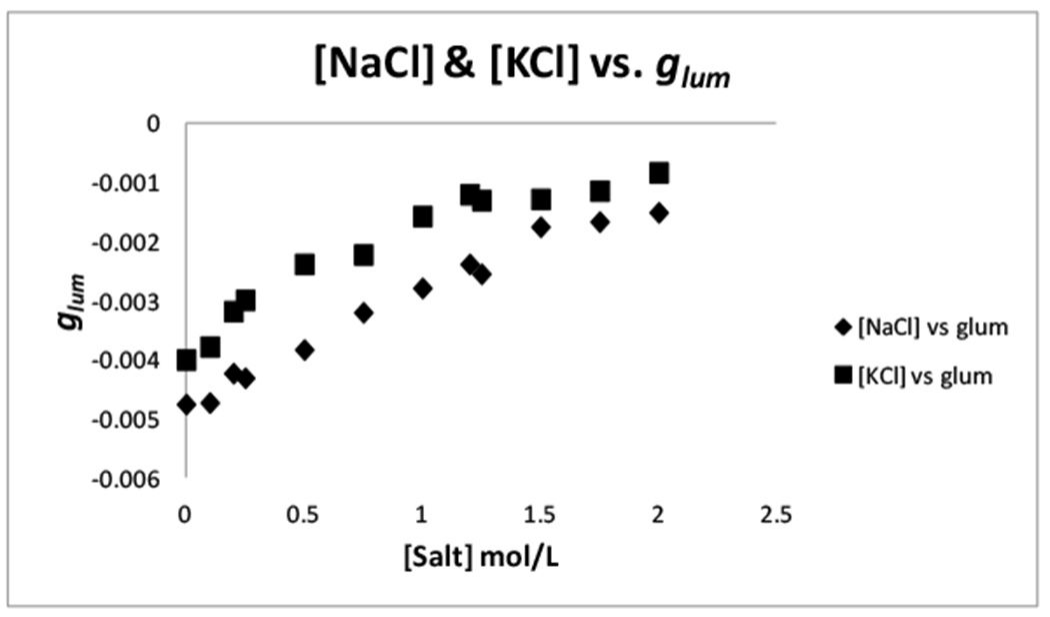
Plot of luminescence dissymmetry values (*g*_*lum*_) of [Tb(DPA)_3_]^3−^. Conditions: 0.005 M [Tb(DPA)_3_]^3−^ with 40 equiv. l-serine and [NaCl] (bottom line triangles, *R*^2^ = 0.9019 or [KCl] (top line circles, *R*^2^ = 0.9774) varied from 0 to 2.0 M, monitored at the spectral range of ^5^D_4_ → ^7^F_5_ at room temperature and pH 6.6, and measured at λ_ex_ = 283.00–289.00 nm and λ_em_ = 542.80–543.00 nm.

To further investigate the solvent effects described earlier in this work, we altered the electronic environment of the system by the addition of NaCl and KCl at various concentrations. The pH for the solutions were maintained at or near pH = 6.6. This particular pH was chosen and maintained at or near 6.6 because at higher pH (i.e., 7), precipitates began to form within the solution. Also, at this pH, l-serine is still in its zwitterionic form, and the 1:3 form of the complex is still present in abundance [27,28]. The fact that a *g*_*lum*_ (−0.00703) was obtained with a NaCl concentration of zero, confirms this. The shift in the | *g*_*lum*_ | at a NaCl concentration of zero was a clear indication that the shift in the equilibrium is solely influenced by the addition of l-serine. Upon increasing the NaCl concentration from 0.0 to 1.49 M, the | *g*_*lum*_ | value became less negative (−0.00283). The | *g*_*lum*_ | value continued to decrease (−0.00173) as the NaCl concentration was increased to a final concentration of 2.0 M. Figure 11 (bottom plot) shows that as the NaCl concentration was increased to 2.0 M, the | *g*_*lum*_ | value closely approached zero. This was an indication that with the addition of NaCl the proximity of the amino acid to the complex may be decreased by the interference of the salt ions. Another explanation was the in addition to the NaCl ions creating an electrostatic screen, the salt ions also decreased the overall charges of the complex (3−) and the amino acid functions (i.e., NH_3_^+^ and COO^−^). By decreasing the overall charge of the complex and the amino acid functional groups (NH_3_^+^, COO^−^) the attraction between the amino acid and the complex is weakened and the observed | *g*_*lum*_ | value decreases. This could be a simple lowering of the electrostatic attraction between the charged surfaces of the hydration shells surrounding the complex and the amino acid, due to the presence of oppositely charged ions.

Without NaCl, or rather, at a salt concentration of zero, the amino acid is free to interact with the outer-sphere (which includes the hydration shells) of the complex. The electrostatic association occurs without further “dampening” or shielding, caused by the salt ion screen. The degree of perturbation can be considered as a function for how close the amino acid is able to initially approach the complex through an initial (primary) electrostatic attraction. This initial attraction initiates the necessary rearrangement of water molecules in the primary solvation sheath of the complex. Any hindrance, such as the presence of salt ions, of this initial attraction hinders the “correct” movement of the primary solvation sheath.

If there was a complete displacement of the solvation sheaths of the amino acid or the complex, they would interact directly; this would result in the observation of a larger | *g*_*lum*_ | value, which is yet to be observed. The degree of perturbation is dependent upon how close the chiral species is able to initially approach the solvation sheath of the complex. The proximity of the chiral species to the solvation sheath of the complex mitigates the strength of the association and also the efficiency of any chirality transfer that may occur from the complex to the solvation sheath and to l-serine. If the chiral species was hindered from approaching the solvation sheath of the complex, any secondary interactions that may also occur (i.e., additional hydrogen bonding interactions or other forces that aid in the stabilization and formation of a stable [Tb(DPA)_3_]^3−^:AA adduct) are not possible, and the resultant | *g*_*lum*_ | should reflect this.

It could also be argued that the amino acid-charged functions, being ionic in nature, are stabilized by the presence of the NaCl ions. The stabilization, then, should result in stronger associations between the amino acid and the complex, therefore forming more stable adducts. This would be evidenced in a larger | *g*_*lum*_ | value (better perturbation) as the NaCl concentration is increased. This experiment, however, shows the opposite. The | *g*_*lum*_ | value in this experiment approaches zero as the NaCl concentration increased to 2.0 M, which was evidence that there was less of a perturbation not an increase. Similar results were observed with the salt KCl as well (Figure 11). The salt KCl (Figure 11 top plot) was added in order to see whether similar results were observed with a similar salt as NaCl. Both salts gave very similar | *g*_*lum*_ | values at each concentration, which indicates that the perturbation of the complex equilibrium is similar with both salts. Since KCl had comparable results to that of NaCl, the conclusions made for NaCl in the discussion above can be further confirmed. The results of the NaCl and KCl studies are important, as they serve to add validity to the notion that the solvation sheaths of the complex are indeed intact and that they mitigate the discriminatory interactions between the complex and the added CEC*.

### 2.8. Preliminary Results with L-Proline

Similar solvent experiments were done with l-proline and will be discussed here briefly. The alcohol solvents were first to be studied with l-proline, and the results are shown in Table 8. It is evident that the results were not comparable to the results with the same solvents where l-serine was used as the CEC*. The only similarities were with methanol, ethanol, and isopropyl alcohol in that they all perturbed the complex equilibrium more than a solution with only water present as the solvent. There were solubility issues when *t*-butyl alcohol was used as the secondary solvent so it was not included in the data. The formamide solvents along with acetone, DMSO, THF, and 1,4-dioxane were also included in this study as well but they all caused less of a perturbation than when l-serine was used as the CEC*. Acetonitrile, chloroform, and glacial acetic acid were excluded from the studies with l-proline, as there were also solubility issues with l-proline and these solvents.

There are significant structural differences between l-serine and l-proline (Scheme 1), which may explain the differences that were observed in the two studies, the most significant being that l-proline is a ring structure and that l-serine is not. The amino group of l-proline is hindered and restricted from rotating freely as the amino group of l-serine, because it is involved in the ring system. Since the amino group of l-proline is restricted from rotating, the negatively charged carboxylate group cannot be rotated away from the negative cloud produced by the water molecules making up the solvation sheath of the complex. It is likely that l-proline may experience repulsive forces (negative charge repulsion) that position l-proline at a distance that is further away from the complex, compared to l-serine, and so there is less of a perturbation when l-proline is the CEC*. As a result, a larger perturbation is observed when l-serine is the CEC* as it has the ability to more freely rotate the negatively charged carboxylate away from any unfavorable interactions with the negative character of the solvation sheath of the complex. Comparing the | *g*_*lum*_ | value of a solution with 0.005 M [Tb(DPA)_3_]^3−^ 40 equiv. l-serine with the corresponding system with l-proline in water as the only solvent (| *g*_*lum*_ | = 0.00238 l-proline vs. | *g*_*lum*_ | = −0.00580 l-serine), it seemed likely that there was repulsion between the complex and l-proline, due to the restricted rotation caused by the l-proline hydrocarbon ring.

Another logical conclusion to make regarding the significant differences seen with l-proline is that the presence of the hydrocarbon ring and the lack of other hydrogen bonding sites on the ring of l-proline would make l-proline much less soluble in the organic solvents than l-serine. If this is true, l-proline may still be able to perturb the equilibrium by interacting with the organic solvents present in the solution through hydrophobic interactions with these solvents as did l-serine. The hydrocarbon ring of l-proline would effectively force the organic solvents closer to the solvation sheath of the complex. This interaction would force the organic solvent molecules closer to the water molecules in the solvation sheath of the complex, causing it to contract in the manor described previously for l-serine. The fact that we still observed a *g*_*lum*_ activity for solutions of l-proline with varying organic solvents may be an indication that this is indeed the case.

Additional studies with l-proline are needed to better understand how l-proline effects the solvent sheath of the complex; although we do know that it is affected from the preliminary results, the extent of what remains to be studied. Solvents that l-proline is more soluble in and that are still miscible with water, will need to be explored in order to study this further. However, the preliminary study with l-proline indicates that the findings and theories proposed for l-serine are more likely to be true. There is an intact solvation sheath around the complex, and as well as the amino acid, the interactions appear different, but the structure of the CEC* has to be taken into account as well. Different molecules will be solvated by solvation sheaths unique to their structures which will likely effect how they interact with another molecule in solution, such as the [Tb(DPA)_3_]^3−^ complex in this case. Since l-proline has a vastly different structure than l-serine, it would not be expected to interact with the complex in quite the same way, which may be indicated by the results presented here. Further study with l-proline and as well as other amino acids with differing structures should be investigated to the extent of l-serine in this work. This would offer a much better understanding of how the structure of the CEC* effects the perturbation, and the possible chirality transfer that may occur between the complex and the solvation sheath solvating the complex.

The preliminary studies with l-proline seem to indicate that the original hypothesis regarding the importance of solvation sheaths is confirmed. It also adds further evidence that the Pfeiffer mechanism is a highly complicated one and cannot be fully described with attributing observations to one variable, such as the dielectric constant of the solvent. The studies with l-proline also add substantial support for the proposed theory that chirality transfer mechanisms should also be included to better understand/describe the functioning of the Pfeiffer mechanism.

## 3. Materials and Methods

The starting materials consisting of terbium(III) and europium(III) chlorides, DPA, l-serine, l-proline, NaCl, and KCl were purchased from commercial chemical distributers (Aldrich, (St. Louis, MO, USA), Bachem (Torrance, CA, USA), or Acros Organics (Thermo Fisher Scientific, Rochester, NJ, USA)) [51] and used without further purification. The organic solvents, MeOH, EtOH, *t*-butyl alcohol, isobutyl alcohol, acetone, DMSO, acetonitrile, formamide, *N*-methyl formamide, *N*,*N*-dimethyl formamide, tetrahydrofuran, 1,4-dioxane, and glacial acetic acid were all dried over 4.0 Å molecular sieves for a 24 h. period before use, in order to remove excess water. The Ln(III) content of stock solutions was determined by titrations with a standardized solution of EDTA in the presence of 0.1 M ammonium acetate and aqueous arsenazo(III). The final concentration for [Tb(DPA)_3_]^3−^ and [Eu(DPA)_3_]^3−^ were 0.005 M with a 1:3.5 ratio of Ln(III):DPA. The concentrations of the amino acids l-serine and l-proline for each experiment were held constant at 40 equiv. (40 equivalents of amino acid relative to 0.005 M [Ln(DPA)_3_]^3−^). The percentages of organic solvents were varied relative to water in ratios of H_2_O:solvent from 90:10 to 50:50. Concentrations of NaCl and KCl were varied from 0 M to 2.0 M. The pH of the solutions was adjusted with aliquot amounts of concentrated NaOH or HCl accordingly. The pH range was reached using the respective aliquot amounts of NaOH and HCl. The solutions were then allowed to sit stirring for a 24 h period. The pH was tested again before running all measurements to ensure the pH was stabilized within the range desired. All solutions were maintained at a pH of 7, or 6.6 in the case of the NaCl and KCl studies.

Circularly polarized luminescence (CPL) and total luminescence spectra were recorded on instrumentation described previously [55]. In short, the instrumentation was equipped with a 1000 W xenon arc lamp from a Spex FluoroLog-2 spectrofluorometer (Horiba Scientific, Edison, NJ, USA), with excitation and emission monochromators of dispersions 4 nm/mm (SPEX, 1681B). The maximum excitation wavelength was determined by running an excitation scan monitoring at λ_em_ = 545.00 nm and corresponding to the ^5^D_4_ ← ^7^F_5_ (Tb) transition. The maximum emission wavelength was then determined by running an emission scan at the maximum λ_exc_. It is common to report the degree of CPL in terms of the luminescence dissymmetry factor *g*_*lum*_ (λ), which is defined as:

(4) $$g_{\mathrm{lum}}=\frac{\frac{\Delta I}{1}}{2}I=\frac{\mathrm{IL}-\mathrm{IR}}{1∕2(\mathrm{IR}+\mathrm{IR})}$$

where *I*_L_ and *I*_R_ refer, respectively, to the intensity of the left and right circularly polarized emissions. The standard deviation, σ_d_, in the measurement of the luminescence dissymmetry factor, *g*_*lum*_, is defined as:

(5) $$\sigma_{d}=\sqrt{\frac{2}{N}}$$

where *N* is the total number of photon-pulses that are counted. All *g*_*lum*_ values were recorded at the maximum λ_exc_ and λ_em_ values. CPL spectra were measured at the maximum λ_ex_ value, with λ_em_ ranging from 530.00 nm to 565.00 nm. ^5^D_0_ ← ^7^F_0_ (Eu) excitation measurements for [Eu(DPA)_3_]^3−^ were measured using a Coherent—599 tunable dye laser (Coherent, Santa Clara, CA, USA) (0.03 nm resolution) with a Coherent Innova Sabre TMS 15 as a pump source. The laser dye used in the measurements was rhodamine 6G dissolved in ethylene glycol. The calibration of the emission monochromator (and subsequently the dye laser wavelength) was performed by passing scattered light from a low power He–Ne laser through the detection system. The error in the dye-laser wavelength is assumed to correspond to the resolution of the emission monochromator (0.1 nm). The optical detection system consisted of a focusing lens long-pass filter and 0.22 m monochromator. The emitted light was detected by a cooled EMI-9558B photomultiplier tube operating in photon-counting mode. All measurements were performed in a quartz cuvette with a path length of 1.0 cm.

## 4. Conclusions

This work presents a detailed and complicated description of the mechanism of the Pfeiffer effect. From this work it is apparent that the effect was highly complicated and could not be described with just one contributing factor, such as the dielectric constant of the solvent. We were able to show that the dielectrics of the solvent environment are much less important than the overall structure of the secondary solvent and the CEC*s that are introduced to solutions containing [Ln(DPA)_3_]^3-^ complexes. The presence of hydrophobic substituents on the solvents that were included in this work were necessary to cause a significant perturbation in the complex equilibrium. Hydrophobic groups are necessary in order to enhance the chiral environment of the lanthanide(III) ion by influencing the solvation sheath of the complex to rearrange or pack closer around the ligand blades. Solvent packing around the ligand blades of the complex may be the key to better understanding and description the Pfeiffer mechanism. The packing or rearrangement of the solvation sheath of the complex may also lead to a secondary source of chirality that is caused by the helical arrangement of the DPA ligands, which is then translated to and retained by the solvation sheath of the complex. It may be that through the chiral solvation sheath that the CEC* is able to preferentially perturb the complex equilibrium, as it would follow that the solvation sheath would also have either a right or left handed helical arrangement. This is dictated by the direction the DPA ligands wrap around the lanthanide(III) ion at the center of the complex. One could also make the conclusion that the CEC*, being chiral, will also have a chiral solvation sheath; however, this may be more transient with the CEC*. Future work dedicated to understanding the Pfeiffer mechanism should include a more in-depth study of the nature of the solvent environment. The chirality transfer mechanism from the complex to the solvation sheath should also be included and understood fully. With the results and observations that are described in this extensive work, it seems more likely that chirality transfer mechanisms may in fact be the key to finally fully understanding the Pfeiffer effect and how it truly functions in solution. In addition to offering a better description of the Pfeiffer effect, understanding the chirality transfer mechanism, may also be important when designing new chiral probes that may have use in a variety of applications.

The theories presented within this article, particularly with those concerning the contraction of the solvation sheath of the complex, are speculative at best. There is, to date, no significant work or literature that is dedicated to the full investigation or description of such a phenomenon applied or described for Ln(III) systems, such as those that are mentioned in this article, to the best of our knowledge. More extensive study is necessary, in order to either refute or support the theories proposed. It is the ultimate goal of this article to open discussion and inspire more extensive study into the solvent behavior of lanthanide(III) systems that exhibit Pfeiffer activity. It is also the goal of this article to draw attention to the lack of meticulous consideration and information available in regards to the solvent environment, and its extreme importance to these systems and similar systems that function with chiral components. At this juncture, it is pertinent to mention that the mechanism of the Pfeiffer effect is highly complex and it cannot be simply described in the context of a singular factor. It is much more complicated than that, as insinuated by the preliminary findings described in this study. In order to offer a more accurate description and understanding of how this mechanism truly operates, it is necessary to open the door for more discussion and investigation into the solvation environment behavior and the impact/importance that it has on the mechanisms of systems such as these.

## Figures and Tables

**Table 1.** Solvents used in the study.

Solvent ^a^ (Pure/Ideal)	(*ε*)	Solvent (Pure/Ideal) ^a^ (*ε*)
1,4-dioxane	2.2	Methanol 33.1
Chloroform	4.8	Acetonitrile 37.5
Glacial acetic acid	6.2	*N,N*-dimethylformamide 38.0
THF	7.6	DMSO 48.9
*t*-butyl alcohol	12.4	H_2_O 80.0
Isopropyl alcohol	19.9	Formamide 109.0
Acetone	21.0	*N*-methylformamide 182.0
Ethanol	24.3	-

a Arranged by increasing ideal dielectric constant.

**Table 2.** Luminescence dissymmetry ratio values (*g*_*lum*_) in the spectral range of the ^5^D_4_ → ^7^F_5_ for 0.005 mol/L [Tb(DPA)_3_]^3−^ with 40 equiv. l-serine, and varying ratios of formamide, *N*-methyl formamide, and *N*,*N*-dimethyl formamide.

*g_lum_* for Each H_2_O:Solvent Ratio ^a^						

Solvent	Ratio: 0	10	20	30	40	50 σ_d_ = ±0.0003
Formamide	−0.00581	−0.00406	−0.00440	−0.00435	−0.00408	−0.00242
NMF	−0.00581	−0.00491	−0.00466	−0.00492	−0.00344	−0.00381
DMF	−0.00581	−0.00646	−0.00781	−0.01234	−0.01835	−0.02037

a Conditions: 0.005 M [Tb(DPA)_3_]^3−^ with 40 equiv. l-serine. Ratios of 90:10 to 50:50 H_2_O:formamide solvent. Monitored at spectral range of the ^5^D_4_ → ^7^F_5_ at room temperature and pH 7.00. Measured at λ_ex_ = 283.00–289.00 nm and λ_em_ = 542.80–543.00 nm.

**Table 3.** Luminescence dissymmetry ratio values (*g_lum_*) for H_2_O:alcohol solvents.

*g_lum_* for Each H_2_O:Solvent Ratio ^a^						

Solvent	Ratio: 0	10	20	30	40	50 σ_d_ = ±0.0003
MeOH	−0.00581	−0.00651	−0.00759	−0.00939	−0.01073	−0.01455
Isopropyl	−0.00581	−0.00743	−0.01072	−0.01460	−0.01583	−0.01911
*t*-butyl	−0.00581	−0.00768	−0.01049	−0.01572	−0.01999	−0.02031
EtOH	−0.00581	−0.00900	−0.01148	−0.01414	−0.01553	−0.02192

a Conditions: 0.005 M [Tb(DPA)_3_]^3−^ with 40 equiv. l-serine. Ratios of 90:10–50:50 H_2_O: alcohol. Solvent, monitored at the spectral range of ^5^D_4_ → ^7^F_5_ at room temperature and pH 7.00, measured at λ_ex_ = 283.00–289.00 nm and λ_em_ = 542.80–543.00 nm.

**Table 4:** Luminescence dissymmetry ratio values (*g_lum_*) for varying ratios of H_2_O:THF and 1,4-dioxane.

*g_lum_* for Each H_2_O:Solvent Ratio ^a^						

Solvent	Ratio: 0	10	20	30	40	50 σ_d_ = ±0.0003
1,4-diox	−0.00581	−0.00735	−0.01358	−0.01739	−0.02206	−0.02819
THF	−0.00581	−0.01189	−0.01072	−0.01699	−0.02140	−0.02611

a Conditions: 0.005 M [Tb(DPA)_3_]^3−^ with 40 equiv. l-serine. Ratios of 90:10–50:50 H_2_O:solvent, monitored at spectral range of the ^5^D_4_ → ^7^F_5_ at room temperature and pH 7.00, measured at λ_ex_ = 283.00–289.00 nm and λ_em_ = 542.80–543.00 nm.

**Table 5.** Luminescence dissymmetry ratio values (*g_lum_*) for varying ratios of H_2_O:DMSO, acetone, and acetonitrile.

*g_lum_* for Each H_2_O:Solvent Ratio ^a^						

Solvent	Ratio: 0	10	20	30	40	50 σ_d_ = ±0.0003
DMSO	−0.00581	−0.00738	−0.01263	−0.01295	−0.02206	−0.01878
acetone	−0.00581	−0.00838	−0.01217	−0.01616	−0.02274	−0.02697
acetonitrile	−0.00581	−0.00926	−0.01073	−0.01526	−0.02140	−0.01515

a Conditions: 0.005 M [Tb(DPA)_3_]^3−^ with 40 equiv. l-serine. Ratios of 90:10–50:50 H_2_O:solvent. Monitored at spectral range of the ^5^D_4_ → ^7^F_5_ at room temperature and pH 7.00. Measured at λ_ex_ = 283.00–289.00 nm and λ_em_ = 542.80–543.00 nm.

**Table 6.** Luminescence dissymmetry ratio values (*g_lum_*) for [Tb(DPA)_3_]^3^− with varying ratios of H_2_O:glacial acetic acid (GAA).

*g_lum_* for Each H_2_O:Solvent Ratio ^a^						

Solvent	Ratio: 0	10	20	30	40	50 σ_d_ = ±0.0003
GAA	−0.00581	−0.00152	−0.00023	−0.00036	−0.01606	−0.03597

a Conditions: 0.005 M [Tb(DPA)_3_]^3−^ with 40 equiv. l-serine. Ratios of 90:10–50:50 H_2_O: GAA, monitored at the spectral range of ^5^D_4_ → ^7^F_5_ at room temperature and pH 7.00, and measured at λ_ex_ = 283.00–289.00 nm and λ_em_ = 542.80–543.00 nm.

**Table 7.** Luminescence dissymmetry ratio values (*g_lum_*) for [Tb(DPA)_3_]^3−^ with varying ratios of H_2_O:chloroform.

*g*_*lum*_ for Each H_2_O:Solvent Ratio ^a^						

Solvent	Ratio: 0	10	2O	30	40	50 σ_d_ = ±0.0003
chloroform	−0.00581	−0.00622	−0.00550	−0.00774	−0.00759	−0.00741

a Conditions: 0.005 M [Tb(DPA)_3_]^3−^ with 40 equiv. l-serine. Ratios of 90:10−50:50 H_2_O:chloroform, monitored at the spectral range of ^5^D_4_ → ^7^F_5_ at room temperature and pH 7.00, and measured at λ_ex_ = 283.00–289.00 nm and λ_em_ = 542.80–543.00 nm.

**Table 8.** Luminescence dissymmetry ratio values (*g_lum_*) for [Tb(DPA)_3_]^3^− with 40 equiv. l-Proline in varying ratios of H_2_O:organic solvent.

**CHCl**_3_	0	10%	50%	30%	40%	50%
*g*_*lum*_ **Ave**	0.00238	0.00159	0.00127	0.00153	0.00177	0.00000

**THF**	0	10%	50%	30%	40%	50%
*g*_*lum*_ **Ave**	0.00238	0.00103	0.00163	0.00248	0.00104	0.00000

**DMSO**	0	10%	50%	30%	40%	50%
*g*_*lum*_ **Ave**	0.00238	0.00069	0.00088	0.00063	0.00059	0.00026

**1,4 Dioxane**	0	10%	50%	30%	40%	50%
*g*_*lum*_ **Ave**	0.00238	0.00065	0.00036	0.00050	0.00068	0.00107

**Acetone**	0	10%	50%	30%	40%	50%
*g*_*lum*_ **Ave**	0.00238	0.00074	0.00116	0.00100	0.00141	0.00110

**Formamide**	0	10%	50%	30%	40%	50%
*g*_*lum*_ **Ave**	0.00238	0.00027	0.00001	−0.00002	0.00001	−0.00017

**NMF**	0	10%	50%	30%	40%	50%
*g*_*lum*_ **Ave**	0.00238	0.00031	0.00018	0.00004	0.00027	−0.00011

**DMF**	0	10%	50%	30%	40%	50%
*g*_*lum*_ **Ave**	0.00238	0.00112	0.00108	0.00264	0.00142	0.00075

**ISOPRO**	0	10%	50%	30%	40%	50%
*g*_*lum*_ **Ave**	0.00238	0.00192	0.00455	0.00803	0.00961	0.00830

***t***-**Butyl**	0	10%	50%	30%	40%	50%
*g*_*lum*_ **Ave**	0.00238	0.00112	0.00236	0.00188	0.002	0.00464

**MeOH**	0	10%	20%	30%	40%	50%
*g*_*lum*_ **Ave**	0.00238	0.00362	0.00447	0.00426	0.00686	0.00771

**EtOH**	0	10%	50%	30%	40%	50%
*g*_*lum*_ **Ave**	0.00238	0.00413	0.00577	0.00683	0.00785	0.00992

Conditions: 0.005 M [Tb(DPA)_3_]^3−^ with-40 equiv. l-proline. Ratios of 90:10–50:50 H_2_O:organic solvent, monitored at the spectral range of ^5^D_4_ → ^7^F_5_ at room temperature and pH 7.00, and measured at λ_ex_ = 283.00–289.00 nm and λ_em_ = 542.80–543.00 nm.
